# Natural history of ovarian cancer

**DOI:** 10.3332/ecancer.2014.465

**Published:** 2014-09-25

**Authors:** Arturo Novoa Vargas

**Affiliations:** Mexican Institute of Social Security (IMSS), Reforma 476, Cuauhtémoc, Juárez, 06600 Distrito Federal, México City, México

**Keywords:** ovarian cancer, natural history

## Abstract

Ovarian cancer is a disease laden with paradigms, and it is a serious health problem. It is important to know its natural history, as it is multifactorial in origin, and also to understand its behaviour given its risk factors which can lead to death from metastasis in patients. It continues to be a challenge for oncologists. An analytical literature review was performed to update the latest concepts of its origin, evolution, risk factors, pre-clinical horizon, and its clinical manifestations; until the death of the patient.

## Introduction

Ovarian cancer is the sixth most common tumour in women. More than 200,000 new cases are diagnosed each year worldwide. Each year, it constitutes 4% of all cancers diagnosed in women, and there are 6.6 new cases per 100,000 women per year [1, 2]. Its history has been known scientifically for over 150 years; during this time, its mortality rate has not changed but its incidence has—the former despite treatments, which are highly expensive and sophisticated. In the last two decades there have been only small improvements in the overall survival rate of five years. While the five-year survival rate has steadily increased from 30–50% with the use of cisplatin, in total, there is only a 5% increase, from 20% to only 25%, in women with advanced tumours. In Europe, more than a third of women with ovarian cancer live for five years after diagnosis [3]. The poor survival rate is related to the diagnosis made in the advanced stages of the disease [4]. It is a neoplasm that responds well to systemic chemotherapy in more than 80% of the cases, when it is administered together with optimal cytoreductive surgery [5]. Despite complete remission (CR) with first-line chemotherapy (CT), ovarian epithelial cancer recurs in over 50% of women.

**Figure 1.**
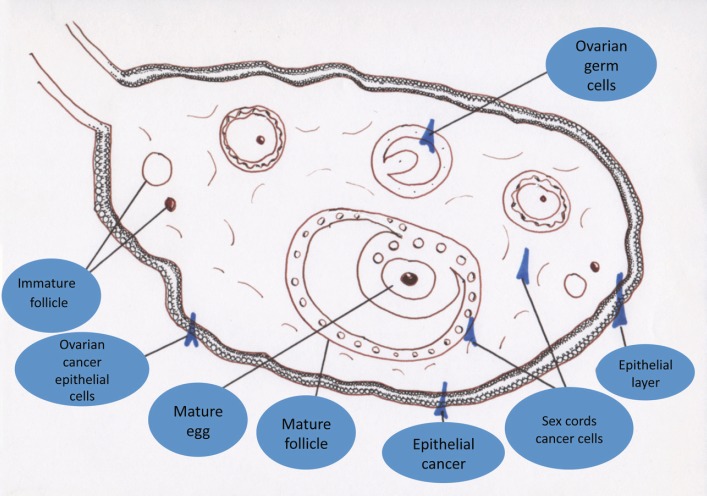
Types of ovarian cancer according to origin.

It is the most common malignancy after breast cancer in women over 40 years of age, particularly in developed countries, and in Mexico it stands in fourth place among the deaths caused by gynaecological tumours [6]. Epithelial ovarian cancer occurs in about 90% of cases, and only 10% originate from germ cells, from sex cords, and from ovarian stroma cells. Approximately 75–80% of epithelial ovarian cases are of the serous histological type. Mucinous, endometrioid, clear cell, Brenner, and undifferentiated lineage cancers are less common (Figure 1) [7].

Most women with ovarian cancer are diagnosed with locally advanced and metastatic disease according to the International Federation of Gynaecology and Obstetrics (FIGO) clinical stages III or IV [8].

## Stages according to FIGO

### Stage III

The tumour involves one or both ovaries, with positive peritoneal implants outside the pelvis, or retroperitoneal, or inguinal positive nodes. There is superficial liver metastasis. The tumour is limited to the true pelvis area, but with a histologically verified malignant extension to the small intestine or omentum.

### Stage IV

The tumour invades one or both ovaries with distant metastasis. There is pleural effusion fluid, positive by histology, and liver parenchymal disease.

## Development

Its origin has not really been clarified, and there are multiple causes, which has led to a number of medical publications in indexed and non-indexed journals. Ovarian cancer is a disease that belongs to the group of chronic and degenerative diseases with extensive natural history. After ten years of control, it can re-occur, with fatal relapses in a short amount of time. Perhaps the critical period to establish a good survival prognosis and a long disease-free period is the first two years of diagnosis, in locally advanced stages. Unlike breast cancer, it appears in two periods of life, the first one being in youth, and the second one postmenopausal.

Epithelial ovarian cancer, the most frequent, occupies third place in the list of gynaecological malignancies worldwide. In 2008, half of new cases were presented in highly developed countries [9]. In our country, Mexico, more than 4,000 new cases were registered and it is the fourth most common cause of death in women, its average diagnosis age being between 50 and 70 years, unfortunately in locally advanced stages [10]. Fortunately, 80% of cases responded to primary treatment, although they have a high relapse rate in 60–70% of cases as a result of a lack of suitable research methods, favoured by the low symptomatology, and a lack of early-stage screening techniques, which hinder timely diagnosis of the only curable stage of ovarian cancer (the early stage) [6]. In the presence of any pelvic tumour, recommendations have been published to understand the risk of malignancy index for ovarian tumour (RMI I), described by Jacobs in 1990. It uses the results of cancer-125 (CA-125) immunohistochemistry report, the ultrasound (US) report which is expressed with a score from 0 to 3; and menopausal status report: 1 if the woman is premenopausal, and 3 if she is postmenopausal. This index has a sensitivity of 85% and a specificity of 97%. In 1996, Tiangulstad devised a variation to the Jacobs score with his RMI I, naming its malignancy risk index RMI II; which, unlike RMI I, has menopausal values from 1 to 4 and pelvic US from 1 to 4, both with the sum of >200 as the cut-off level [11]. Survival is influenced by tumour volume; the higher the tumour load, the lower the patient’s survival. From 1975 to 2004, with optimal surgery and with the best chemotherapy at the time, a median overall survival rate was reached between two to five years, and the survival after relapse did not exceed two years [12]. Large clinical studies published in the past 15 years reveal that the median progression-free survival (PFS) rate of patients with advanced disease oscillates between 16 and 23 months, while the median secondary cytoreduction (SC) ranges between 31 and 65 months [13]. Following CT with cisplatin, improvement with treatments has been scarce, and survival rates have not improved substantially. With the need for more improved therapeutic regimens, there has been an idea to target the use of anti-angiogenic agents in treatments for epithelial ovarian cancer.

## Heredity of ovarian cancer

Alterations for ovarian cancer are transmitted through mitosis from one cell to another, but are not transmitted from parents to children. There must be a predisposition or susceptibility to germline mutation or an inherited susceptibility for that to happen. The cause and mechanism of hereditary cancer are the fathers’ sperm. Once a person has inherited cancer, the individual becomes a carrier and will transmit it to their offspring according to Mendelian laws, depending on the dominant or recessive nature of the altered gene causing the cancer. Usually, it is dominant if it is an oncogene and recessive if it is a mutated tumour suppressor gene. The best known hereditary cancer is breast cancer. It has this characteristic in 5–10% of the cases. Its main cause is the mutation of the BRCA1 tumour suppressor genes from chromosome 17 or BRCA2 from 13. The first of these genes manifests itself in breast and ovarian cancer, because it is a tumour suppressor gene. In general hereditary transmission is because of mutant allele, which will only manifest itself in daughters, developing into a tumour. The abnormal presence of BRCA1 is associated in women with a 50% to 80% probability of developing breast cancer and a predisposition to ovarian cancer [14].

## Hereditary risks

- BRCA 1 and BRCA 2 mutations: Risk for ovarian cancer from 27–44%, compared to 1% of the general population.
- Breast-ovary syndrome: Risk of 10–44%.
- Li-Fraumeni syndrome.
- Lynch syndrome: Risk of 9–12%.
- Ashkenazi Jewish population: Risk of 16–60%.

**Figure 2.**
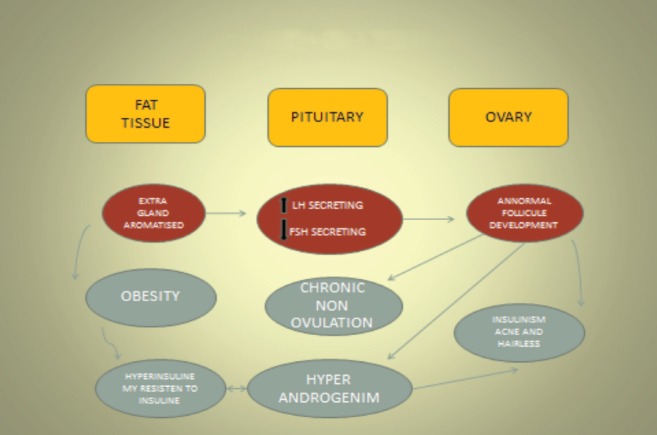
Migration of boundary cells. LH: Luteotropic hormone. FSH: Follicle-stimulating hormone.

The risk factors of epithelial ovarian cancer are typical of perimenopausal and postmenopausal women [15, 16]. Its frequency increases with the number of decades. The most common factors are:

1. Family history of ovarian cancer.
2. Medium-high socioeconomic status, especially in industrialised countries.
3. Nulliparity, infertility, and use of ovulation-stimulating drugs [17].
4. White race.
5. High-fat diet and obesity.
6. Polycystic ovaries (Stein-Leventhal syndrome) [18].
7. Personal history of breast, colon, or endometrial cancer (hormone-dependent tumours).
8. Exposure to asbestos, talc, or radiation.
9. Migration from adjacent cells (see Figure 2) [19].
10. Low concentration of selenium bound to proteins.

Of the latter, the expression of protein-bound selenium in the ovary, there is an overexpression of the epithelial cells of the gonad, compared with overexpression of the neoplastic cells of the ovary. It was less than in normal epithelial cells [20].

## The biochemistry of ovarian cancer

Polycystic ovary syndrome (PCOS) is a heterogeneous genetic disorder, involving metabolic and endocrine problems. It occurs most frequently in women of reproductive potential and is characterised by an increased concentration of androgens circulating in the blood. It has a markedly negative effect on the metabolism of normal bodily functions. PCOS can evolve into metabolic syndrome, affecting the body’s energy utilisation. This takes the form of a markedly high rate of insulin synthesis in the blood, abdominal obesity, increased blood pressure, dyslipidemia, insulin resistance, type 2 diabetes mellitus, cardiovascular disease, and endometrial hyperplasia. Changes can also take place in the regulation of gonadotropin release, and this interferes with the ovarian steroid inhibition-feedback loop. This results in increased secretion of luteinising hormone in the woman’s body. The secretion of follicle-stimulating hormone (FSH) also increases; the stromal cells and ovarian theca cells are hyperactive, and because of this hyperactivity, the secretion of androgens increases; all this allows abnormalities to appear in the biochemistry of the body’s metabolism after menopause. There are various types of pharmacological treatment for PCOS, including lifestyle changes such as reduced dietary intake, physical exercise, controlling nitrosodimethylamine production, its increased metabolism in the ovary, and its reaction with the proteins there. Nitrosodimethylamine is metabolised in the ovaries by the mitochondria and the microsomal enzyme system. The microsomes and mitochondria cause the nitrosodimethylamine in the ovary to degrade and be converted to reactive toxic metabolites and formaldehyde. These become attached to the ovarian proteins with covalent bonds. Most of this degradation takes place in the microsomes. When the DNA of the normal epithelium of the ovary has been separated and analysed by immunohistochemistry tests, it shows changed sequences of the deoxyribonucleic acid bases. These changes result from the toxic effects of the reactive metabolites of the nitrosodimethylamine in the DNA of the normal ovarian epithelial cells. This path can occur in any woman exposed to tobacco or alcohol consumption [21].

## Non-hereditary factors

- Age: >45 years.
- Early menarche: relative risk (RR) 1.0 (95%, confidence interval CI 0.6–1.3).
- Late menopause: RR 2.5 (95% CI 1.1–5.8).
- Nulliparity: Pregnancy reduces the risk of ovarian cancer (OR 0.78 for each full-term pregnancy; nulliparity has a RR of 1.7 (95% CI 1.1–2.8).
- Medical history of breast cancer.
- Infertility treatment with no full-term pregnancy: The use of clomiphene citrate is associated with a RR of 2.3 (95% CI 0.5–11.4) compared with infertile women who do not use it.
- Obesity: RR 2.05 in women with a high BMI >30, aged over 18 years.
- Use of hormone replacement therapy for more than five years: RR 1.5 (95% CI 0.9–2.6).
- Smoking: Odds ratio (OR) 1.0.
- Use of talc and exposure to asbestos: Daily use of talc RR 1.3 (95% CI 0.8–1.9).

## Familial risk factors (9% of cases)

- Two immediate family members with breast or ovarian cancer, including one <50 years at the time of diagnosis: OR 2.90 (95% CI 1.92–4.36).
- One family member with unilateral breast cancer <40 years; bilateral <30 years: OR 1.35 (95% CI 1.03–1.78).

Clearly established protective factors include:

1. More than one pregnancy carried to term.
2. Use of oral contraceptives and breast-feeding, the latter being related to continuous ovulation.
3. Tubal ligation not clearly demonstrated.

Lynch II syndrome includes non-polypoid colorectal carcinoma, endometrial cancer, upper gastrointestinal tract cancer, urothelial cell carcinoma of the renal pelvis, ureteral cancer, and ovarian cancer [22]. Published papers have stated that oral contraceptives can reduce the risk of ovarian cancer—particularly in carriers of BRCA mutations, and also depending on the period of use—by up to 20% for every five years’ oral contraceptive use [24].

## Investigation

To date, there is no suitable programme for detection; diagnostic methods are expensive and do not detect malignant tumours in the early stages of disease. In most cases, women are diagnosed at stages of local progression or in metastatic disease with a poor prognosis for survival. However, in women with hereditary risk factors or those identified during a directed history, it is acceptable to do immunohistochemical tests with tumour markers. We should not forget the “*sine qua non*” investigation for a suspected adnexal tumour: pelvic ultrasound, preferably using colour Doppler, and better still, by the vaginal route in the early stages. The above has not had a statistically significant impact on the prognosis for survival of women with ovarian cancer. Women with high-risk factors who have completed their family must undergo a prophylactic salpingo-oophorectomy before the age of 35 years, though this does not eliminate the possibility of future peritoneal carcinomatosis, similar to advanced ovarian cancer. Depriving menopausal women of hormone replacement therapy has not improved the statistics for survival of ovarian cancer patients, and its absence has even favoured cardiovascular ischaemia and loss of bone mass.

## Diagnosis

In general, ovarian cancer is revealed by increased abdominal volume, but the symptoms are vague and frequently an adnexal tumour is overlooked because the discomfort is not intense and is often confused, by the patient and even by the doctor, with gastrointestinal conditions and conditions misleadingly appearing to originate in the urinary tract. When the malignant ovarian tumour does become apparent, 79% of cases are diagnosed at advanced stages, and symptoms will depend on the organ affected and invaded. At this time the woman experiences intense gastrointestinal symptoms, with lower abdominal or pelvic pain, and periodic constipation and diarrhoea; vaginal bleeding also occurs. The patients are usually treated as if the condition originated from ulcers, colitis, and not infrequently liver disease, mainly disease of the gallbladder. The volume and diameter of the abdomen increases suddenly, through ascites, and this leads to the gradual onset of dyspnoea, in line with the amount in litres that is pressing upwards on the diaphragm and impeding respiratory movement. Unlike with other tumours, the patients’ body weight increases because of the malignant-free fluid in the abdominal cavity. When an ovarian tumour is diagnosed by accident at the early stages, the usual diagnosis is of a benign adnexal tumour, which generally works for most fertile women. This generally regresses at the next menstrual period or the painful symptoms improve. The diagnosis of malignant ovarian tumour can also be made accidentally or incidentally during a gynaecological investigation and sometimes because of twisting of the ovarian pedicle, which requires an investigative laparotomy for acute abdomen, mostly with no indication for oncological surgery. Once the ovarian tumour has been diagnosed as malignant, the prognosis for survival will be related to the following factors:

- Age and menopause status.
- Tumour size.
- Tumour stage.
- Characteristics of the tumour by imaging (ultrasound, CT, MRI).
- Presence or absence of symptoms.
- Tumour marker values (cancer-125, cancer 19.9).
- Unilateral, compared with bilateral.

The doctor should never neglect to take a history directed to adnexal tumour, and carry out a physical examination including digital rectal and vaginal examination, which is essential to increase the possibilities for initial surgery (naturally, performed by an oncologist). The initial assessment of patients with suspected ovarian cancer, following the initial history-taking, physical examination, laboratory results, and tests for tumour marker cancer-125, must focus on investigating the contents of the abdominal cavity. Therefore, imaging studies must be conducted, such as ultrasound CT, MRI, and special techniques of radioactive isotope scanning; none of these give details of the correct staging of the tumour, and at the end of the study an abdominal CT, chest x-ray, and bone scan must be carried out. Where technically possible the recommended first-line ultrasound procedure for a suspected single adnexal tumour is by the vaginal route, using colour Doppler. This has a sensitivity of over 93.5% and specificity of 91.5%. If this is not possible, MRI is the most appropriate for adnexal investigation, particularly if there is suspected tumour activity outside the pelvis. It has a sensitivity of 91.1% and specificity of 84%. CT has shown 87.2% sensitivity and 84% specificity [25]. Serum levels of cancer-125 tumour-associated antigen can be high in other malignant tumours such as: breast cancer, pancreatic cancer, colon cancer, bronchogenic cancer, and endometrial cancer. Therefore, it is not recommended as a single or standard method for diagnosing ovarian cancer; when the result is elevated to three times its normal value, it is considered to have 78.7% sensitivity and 77.9% specificity. Elevated cancer-125 can be found in various benign ovarian disorders, particularly in premenopausal women with disorders related to infertility, such as endometriosis, endometrial cyst, pelvic inflammatory disease, hepatitis, pregnancy, menstruation, peritonitis, and after recent abdominal surgery. Until this investigation finds evidence of disease outside the abdominal cavity, a protocol-based exploratory laparotomy is an essential part of the initial investigation for a patient. Romagnolo, Park, and Desfeux have reported finding no significant difference in the survival of patients with early-stage ovarian cancer or in borderline ovarian tumours [26, 27, 28]. Fertility-preserving surgery is advisable in most adnexal tumours located in an ovary, and with tumours that are borderline, or have a low potential for malignancy, at surgical stage I, for ovarian cancer, as long as this does not affect the patient’s survival.

## Histopathological and molecular diagnosis

The strains from ovarian cancer originate from the epithelial, the germ cells, and the gonadal stroma. From the former derive the serous, mucinous, endometrioid, undifferentiated, clear cell, small cell, and Brenner tumour adenocarcinomas. This group makes up about 45 % of the malignant neoplasms. Five percent (5%) of the endometrioid tumours are generated in endometriosis. It is reported that 25% to 33% of the cases are associated to endometrial cancer. The clear cell ones have the worst survival rate. The epithelial strains are characterised for rising the non-specific tumour markers, significantly the cancer-125 in nonmucinous tumours. In mucinous tumours, the cancer-19.9 increases with higher frequency in a formidable response to both schemes with chemotherapy that includes the platinum derivatives.

The non-epithelial strains, mainly the germ ones show more presence in young menstruating people. The tumour markers are mainly increased by: carcinoembryonic antigen, alpha-fetoprotein, and the chorionic gonadotropin beta fraction; the tumours in ovarian germ cells represent 20% of the neoplasms where only 3% is associated with malignancy. Tumours are a group of heterogeneous and complex neoplasms, and up until now there are no etiological factors that have been defined that may be associated to its presentation, despite the many descriptions of diverse chromosomal alterations (3q27-q28, 12q22, 5q34q35, chromosome 14) and processes regarding DNA repair (short-arm of chromosome 12) that contribute to its development. Gonadal dysgenesis is associated to the growth of dysgerminomas in at least 50% of reported cases and some patients have shown that there is an overexpression of p53 gene [29]. The most aggressive of these is the cancer germinal ovary of endodermal sinus with an astonishing increase of alpha-fetoprotein and beta fraction, as well as malignant neoplasm with an adverse forecast. The dysgerminoma, the most frequent of all germs, does not have a tumour marker but it normally increases the lactate dehydrogenase significantly. Currently, there is the goal of classifying ovarian malignant neoplasms according to their morphological and molecular structure, thus grouping the different variations under type I and type II (refer to Table 1).

Immature teratomas show clinically as solid tumours having isolated areas with fatty deposits and calcifications. Yolk sac tumours normally show as tumours with mixed solid and cystic areas. The capsular rupture or bright punctate comes about as a result of an increase in vascularity and because of the creation of small vascular aneurysms. The embryonal carcinoma and the polyembryoma rarely show in their pure form. Instead, they become a part of the mixed tumours of germ cells (see Table 2) [30].

The papillary serous carcinoma are better classified as high-grade and low-grade (Table 3). The method used is the one suggested by MD Anderson, based on the mitosis number and the nuclear pleomorphism [31]:

- **Low-grade:** Mild-to-moderate pleomorphism<12 mitosis x 10 fields of 400x.
- **High-grade:** Significant pleomorphism with more than 12 mitosis x 10 fields of 400x.

Currently, there are no molecular markers that identify groups associated with the forecasted results. Among the related factors, these are some of them: tumour heterogeneity, size of the studied population, and the different sample processing techniques [6].

## Notes

Ovarian cancer will keep on being a high-risk threat to women, given that its diagnosis in initial stages yields a very low percentage. Therefore, the majority of patients will die in spite of today’s treatments. Nowadays there is no low-cost effective method to perform the appropriate screening. I believe it is necessary for every woman after menarche to undergo a pelvic US as a research study. Those with hereditary risk factors of ovarian tumours must have screening tests with immunohistochemical proofs, such as the commonly used tumour markers panel, to aid their diagnosis. It is imperative to remember that ovarian cancer can only be cured in stage I (Table 4), provided there is no bad forecast.

## Conclusions

Ovarian cancer does not present with an effective research method. Good tools for early diagnosis of high-risk tumours are the ultrasound as a *sine qua non* image and immunohistochemistry studies. The natural history of ovarian cancer indicates that it will remain as the tumour that takes the life of women in reproductive age for several decades to come.

## Figures and Tables

**Table 1.** A classification of ovarian tumours based on genetic mutations and biological behaviour.

TYPE I	TYPE II
Low-grade papillary serous	High-grade papillary serous
Endometrioid grade I and II	Endometrioid grade III
Mucinous tumours	Undifferentiated carcinoma and mixed Müllerian tumour

**Table 2.** The main differences between epithelial tumours and malignant ovarian germ tumours.

Characteristics	Epithelial tumours	Cell tumours germs
**Prevalence**	Much more common	Less common
**Age group**	Mainly adult women^*^	Mainly female children and young women
**Affected race**	More common in white women	More common in black and Asian women
**Laterality**	Malignant serous and mucinous tumours, clear cells and endometrioid; they are bilateral in 57.5%, 21.3%,13.3%, and 26.8% of the patients, respectively.	Usually bilateral, but can be bilateral in 4.3% of the patients.
**Tumour Markers**	cancer-125	a-fetoprotein and b-hCG
**Endocrine manifestations**	No	Resulting from hormone production
**Frequency of five-year survival**	The global from the 44% depends on the stage of the tumour	100% for dysgerminomas and 85% for other types of germ tumours.

* 50% of all cases occur in adult women of >65 years old.

**Table 3.** A comparison of low-grade and high-grade tumours.

Grade	KRAS/BRAF	TP53	Presentation age	Response to platinum
Low	Muted	Native	Aprox. 43 years old	Resistant
High	Native	Muted	Aprox. 63 years old	Sensitive

**Table 4.** Frequency distribution of five-year survival during clinic stage.

Clinic stage	Distribution (%)	Five-year survival
I	20	90
II	10–5	80
III	45	20–30
IV	15	<5
